# Molecular Characterization of *Candida parapsilosis* by Microsatellite Typing and Emergence of Clonal Antifungal Drug Resistant Strains in a Multicenter Surveillance in China

**DOI:** 10.3389/fmicb.2020.01320

**Published:** 2020-06-16

**Authors:** Li Zhang, Shu-Ying Yu, Sharon C.-A. Chen, Meng Xiao, Fanrong Kong, He Wang, Ya-Ting Ning, Min-Ya Lu, Tian-Shu Sun, Xin Hou, Meng-Lan Zhou, Wei Kang, Ge Zhang, Si-Meng Duan, Ying-Chun Xu

**Affiliations:** ^1^Department of Laboratory Medicine, Peking Union Medical College Hospital, Chinese Academy of Medical Sciences, Beijing, China; ^2^Beijing Key Laboratory for Mechanisms Research and Precision Diagnosis of Invasive Fungal Diseases (BZ0447), Beijing, China; ^3^Graduate School, Peking Union Medical College, Chinese Academy of Medical Sciences, Beijing, China; ^4^Centre for Infectious Diseases and Microbiology Laboratory Services, ICPMR – Pathology West, University of Sydney, Westmead, NSW, Australia; ^5^Medical Research Center, Peking Union Medical College Hospital, Chinese Academy of Medical Sciences, Beijing, China

**Keywords:** *Candida parapsilosis*, molecular epidemiology, microsatellite typing, cluster, antifungal resistance

## Abstract

*Candida parapsilosis* is an important species causing invasive candidiasis (IC) in China. The present survey was a national multicenter study of the molecular epidemiology and antifungal susceptibility profiles of *C. parapsilosis*. Non-duplicate *C. parapsilosis* isolates were collected from 10 hospitals across China in the CHIF-NET program 2016–2017. Isolates were genotyped using four highly polymorphic microsatellite markers, and susceptibility profiles determined using Sensititre YeastOne^TM^ YO10. A total of 319 *C. parapsilosis* from separate patients with IC were studied; 49.2, 17.9, and 10.3% isolates were from patients in surgical departments, general intensive care units (ICUs) and neonatal ICUs (NICU), respectively. *C. parapsilosis* showed good susceptibility to nine antifungal drugs. Microsatellite analysis identified 122 microsatellite (MT) types. Most MT types had sporadic distribution. However, we identified 32 clusters across 10 hospitals; seven clusters were caused by seven endemic genotypes involving five or more isolates in hospitals designated as H01, H02, H06, and H10. These clusters mainly affected surgical departments and ICUs, except for genotype MT42 which was seen in 22 patients from NICU (hospital H06). Of 16 fluconazole-resistant isolates, seven from hospital H02 shared the same genotype MT70, and three from hospital H04 were of genotype MT47. For 37 isolates with non-wild type MICs to 5-flucytosine, 29 were from hospital H01 (genotype MT48). Here we present the first nationwide molecular epidemiology study of *C. parapsilosis* in China, identified several previously unrecognized clusters, which included antifungal drug resistant isolates. These findings provide important data for control of IC in China.

## Introduction

Invasive candidiasis (IC) is an important fungal disease among hospitalized patients, associated with significant mortality and excessive medical costs (Kullberg and Arendrup, 2015). Although *Candida albicans* still overall accounts for the majority of IC, many studies have reported increased prevalence of infections caused by non-*C. albicans* species (Chow et al., 2008; Silva et al., 2012; Pfaller et al., 2014). Particularly, the incidence of *C. parapsilosis* infection is increasing among hospitalized patients and has become one of the main pathogens causing candidemia in certain geographical areas including China (Guinea, 2014; Pfaller et al., 2014; Puig-Asensio et al., 2014; Strollo et al., 2016). The National China Hospital Invasive Fungal Surveillance Net (CHIF-NET) program is an ongoing nationwide, multicenter surveillance network established in July 2009 to provide data on the epidemiology of invasive fungal infections in China (Wang et al., 2012; Xiao et al., 2015, 2018). In the period from 2010 to 2015, *C. parapsilosis* was the second most common species isolated from bloodstream after *C. albicans* (32.3%), accounting for 28.9% of cases (Xiao et al., 2018).

Of note, during the ongoing program, there was a large increase in the rates of isolation of *C. parapsilosis* in hospital H01 in 2016 (*n* = 47) compared to 2015 (*n* = 7), which alerted us to a potential outbreak of infection in this hospital. *C. parapsilosis* is well established as a cause of nosocomial fungemia outbreaks, particularly in neonatal intensive care units (NICUs) (Hernández-Castro et al., 2010; Miranda et al., 2012; Pammi et al., 2013; Romeo et al., 2013; Magobo et al., 2017; Asadzadeh et al., 2019). Outbreaks have been reported in diverse geographical regions, with most outbreaks occurring in NICUs and ICUs (Garcia et al., 2004; Kuhn et al., 2004; Dizbay et al., 2008; Brillowska-Dabrowska et al., 2009; Hernández-Castro et al., 2010; Vaz et al., 2011; Diab-Elschahawi et al., 2012; Miranda et al., 2012; Pulcrano et al., 2012; Pinhati et al., 2016; Magobo et al., 2017). In addition, there have been reports of clonal transmission involving fluconazole-resistant strains in Brazil and India (Thomaz et al., 2018; Singh et al., 2019). Of the three species of *C. parapsilosis* complex, (*C. parapsilosis* sensu stricto, *C. orthopsilosis*, and *C. metapsilosis*), most data on outbreaks have focused on *C. parapsilosis* sensu stricto (Tavanti et al., 2005). In China, however, there are relatively few descriptions of outbreaks of *C. parapsilosis* and accompanying molecular epidemiology investigations (Wang et al., 2016; Qi et al., 2018).

Based on the unexpected observation of potential case clusters in the CHIF-NET study above, the present study aimed to present the findings of the molecular epidemiology of *C. parapsilosis* sensu stricto isolates obtained from patients from 10 hospitals surveyed between August 2015 and July 2017. We chose to use a four-locus microsatellite-based typing method to genotype *C. parapsilosis* sensu stricto isolates described by Sabino et al. (2010) as this method has proven to be of good discriminatory (power of 99.9%), and is a common tool for genotyping *C. parapsilosis* sensu stricto (Vaz et al., 2011; Romeo et al., 2013; Delfino et al., 2014; Sabino et al., 2015). We used these microsatellite markers to distinguish isolates from all participant hospitals, aiming to uncover the endemic genotypes in different hospitals, and to call genetic association to support epidemiologically linked isolates in case clusters.

## Materials and Methods

### Isolates

A total of 319 non-duplicate *C. parapsilosis* isolates cultured from 319 patients with IC were collected from 10 hospitals in the CHIF-NET program 2016 and 2017 [hospitals H01 (*n* = 98 isolates), H02 (*n* = 22), H03 (*n* = 25), H04 (*n* = 18), H05 (*n* = 19), H06 (*n* = 26), H07 (*n* = 16), H08 (*n* = 36), H09 (*n* = 8), and H10 (*n* = 51)]. The 10 hospitals were located at different districts across China, including Beijing, Fujian, Guangzhou, Henan, Hubei, Jilin, Liaoning, Shaanxi, Shanghai, and Sichuan. Among the 319 patients, there were 24 who had isolates cultured simultaneously from blood as well as from the tip of vascular catheters which had been *in situ.* The 24 strains from the catheter tips were genotyped in addition to the blood isolates, to determine if they shared the same genotype.

All strains were forwarded to a central laboratory (The Department of Clinical Laboratory, Peking Union Medical College Hospital) for confirmation of species identification by using the Vitek MS system (BioMerieux, France) selectively supplemented by ribosomal DNA sequencing as required (Wang et al., 2012; Zhang et al., 2014). The program was approved by the Human Research Ethics Committee of Peking Union Medical College Hospital (S-263).

### Antifungal Susceptibility Testing

*In vitro* antifungal susceptibility to nine antifungal drugs was performed using the Sensititre YeastOne^TM^ YO10 system (Thermo Scientific, Cleveland, OH, United States) following the manufacturer’s instructions. The quality control strains included *C. parapsilosis* ATCC 22019 and *Candida krusei* ATCC 6258. MIC values of anidulafungin, micafungin, caspofungin, voriconazole, and fluconazole were interpreted according to current Clinical and Laboratory Standards Institute (CLSI) M60 clinical breakpoints (CLSI, 2017). Where there are no CLSI breakpoints, species-specific epidemiology cutoff values (ECVs) were used to define isolates as wild type (WT) or non-wild type (NWT) for amphotericin B, posaconazole, itraconazole, and flucytosine (Pfaller and Diekema, 2012; CLSI, 2018).

### Microsatellite Typing

Genotyping of all *C. parapsilosis* isolates was performed using a panel of four highly polymorphic microsatellite markers as described by Sabino et al. (2010), namely B5, CP1, CP4, and CP6. Amplification reactions and allele sizes analysis were performed as previously reported (Wang et al., 2016). The genetic relationships between the genotypes were studied by constructing a minimum spanning tree using the BioNumerics software v7.6 (Applied Maths, Sint-Martens-Latem, Belgium), treating the data as categorical information. Genotypes showing the same alleles for all four markers were considered identical. Endemic genotypes were defined as genotypes infecting ≥2 different patients in one hospital. A cluster was defined as a group of ≥2 patients infected by an endemic genotype (Escribano et al., 2013).

## Results

### Clinical Characteristics of Patients

The characteristics of patients and of *Candida* species at the different hospitals are shown in Table 1. For the 319 isolates, 72.7% were cultured from male patients, and 27.3% from female patients. Cases of IC included candidemia (*n* = 230), abdominal *Candida* infection (*n* = 61), central nervous system infection (*n* = 6), and other invasive infections (*n* = 22). Generally, the numbers of isolates increased with age, with 38.6% isolates from patients >60 years. In the present study, 49.2, 11.6, and 17.9% of isolates were collected from patients located in surgical departments, medicine departments and ICUs, respectively; 2.5 and 10.3% isolates were from patients in the pediatric ward and NICUs, respectively.

**TABLE 1:** Clinical characteristics of 319 patients with invasive *C. parapsilosis* infections.

**Items**	**Total no. (%)**	**H01**	**H02**	**H03**	**H04**	**H05**	**H06**	**H07**	**H08**	**H09**	**H10**
**Gender**											
Male	232(72.7)	73	18	17	12	15	17	13	29	5	33
Female	87(27.3)	25	4	8	6	4	9	3	7	3	18
**Age**											
0–5	43(13.5)	0	0	1	7	0	23	1	9	2	0
6–20	4(1.3)	0	1	1	1	0	0	1	0	0	0
21–40	37(11.6)	8	1	4	0	6	0	4	7	2	5
41–60	112(35.1)	31	14	12	7	9	2	6	13	2	16
61–80	115(36.0)	55	4	7	3	3	1	4	7	2	29
>80	8(2.5)	4	2	0	0	1	0	0	0	0	1
**Specimen**											
Blood	230(72.1)	74	13	8	16	13	24	6	20	7	49
Other	89(27.9)	24	9	17	2	6	2	10	16	1	2
**Ward**											
Medicine	37(11.6)	9	3	4	2	7	1	4	2	2	3
Surgical department	157(49.2)	69	9	12	5	3	1	4	12	4	38
Pediatrics	8(2.5)	0	0	0	1	0	0	1	6	0	0
ICU	57(17.9)	13	9	3	4	8	1	5	3	1	10
SICU	16(5.0)	4	0	3	0	1	0	2	6	0	0
NICU	33(10.3)	0	0	1	6	0	23	0	3	0	0
Other	11(3.4)	3	1	2	0	0	0	0	4	1	0
Total	319	98	22	25	18	19	26	16	36	8	51

*ICU, intensive care unit; SICU, surgical intensive care unit; NICU, neonatal intensive care unit.*

### Antifungal Susceptibility of *C. parapsilosis* Isolates

The antifungal susceptibilities of the isolates are shown in Table 2. In general, *C. parapsilosis* was susceptible to fluconazole (94.4% susceptible), voriconazole (95.3% susceptible), posaconazole (WT isolates, 97.5%), and itraconazole (WT isolates, 100%). Sixteen fluconazole-resistant isolates were from hospitals H02 (*n* = 7), H04 (*n* = 3), H08 (*n* = 4), H09 (*n* = 1), and H07 (*n* = 1). All 319 isolates were susceptible to echinocandins and had WT MICs to amphotericin B except for two isolates which were classed as moderately susceptible to micafungin (MIC, 4 μg/mL). For 5-flucytosine, 37 (11.6%) isolates had NWT MICs, largely from hospital H01 (*n* = 32 isolates), and the rest from hospitals H04 (*n* = 3) and H08 (*n* = 2).

**TABLE 2:** Antifungal susceptibility profiles of *C. parapsilosis* isolates (μg/mL).

**Antifungals**	**MIC range**	**MIC50**	**MIC90**	**S/WT**	**R/NWT**	**Breakpoint references**
Anidulafungin	0.03–2	1	2	100	0	CLSI, 2017
Micafungin	≤0.008–4	1	2	99.4	0	CLSI, 2017
Caspofungin	0.12–2	0.5	1	100	0	CLSI, 2017
Flucytosine	≤0.06 – ≥64	≤0.06	64	88.4	11.6	Pfaller and Diekema, 2012
Posaconazole	≤0.008–2	0.03	0.12	97.5	2.5	CLSI, 2018
Voriconazole	≤0.008–4	0.015	0.06	95.3	4.7	CLSI, 2017
Itraconazole	≤0.015–1	0.06	0.12	100	0	Pfaller and Diekema, 2012
Fluconazole	≤0.12–128	0.5	1	94.4	5	CLSI, 2017
Amphotericin B	≤0.12–2	0.5	1	100	0	CLSI, 2018

*S, susceptible; R, resistant; WT, wild type; NWT, non-wild type.*

### Microsatellite Typing (MT) of *C. parapsilosis*

**FIGURE 1:**
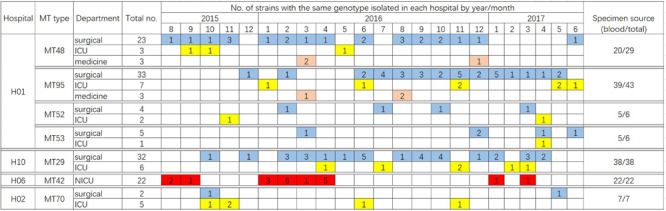
Timeline of the ward distribution of MT types involved in clusters of in the hospitals, of which only clusters with more than five patients infected were shown in the figure. Different colors represent different departments. Blue, surgical department; yellow, ICU; orange, medicine department; red, NICU. In hospital H06, a total of 23 isolates were collected from NICU, of which 22 isolates belonged to the genotype MT42.

A total of 122 MT types for 319 isolates were identified (Supplementary Table S1). There were differences in the discriminatory power of four alleles – allele B5 (*n* = 29, 23.8%), CP1 (23, 18.9%), CP4 (59, 48.4%), and CP6 (60, 49.2%). Among the 122 MT types, 83 genotypes were distributed sporadically. The remaining 29 genotypes were identified within 32 clusters – MT81 formed four clusters in four different hospitals, with the remaining 28 clusters encompassing different genotypes) (see Table 3). In hospital H01, there were seven clusters, involving a total of 91 isolates. In H06 and H10, there were also endemic genotypes involving large number of isolates, like MT29 (*n* = 38) and MT42 (*n* = 22) (Table 3). The sampling interval within each endemic genotype in the same ward differed with a wide range (1–566 days). The detailed timeline and the ward information for seven endemic genotypes involving five or more isolates are shown in Figure 1.

**TABLE 3:** Distribution of 122 MT types in 10 hospitals.

	**H01**	**H02**	**H03**	**H04**	**H05**	**H06**	**H07**	**H08**	**H09**	**H10**
No. of strains	98	22	25	18	19	26	16	36	8	51
No. of MT types	14	12	18	12	13	4	15	30	6	13
No. of strains/No. of MT types	7	1.8	1.4	1.5	1.5	6.5	1.1	1.2	1.3	3.9
Endemic genotypes	MT95 (43)	MT70 (7)	MT81 (4)	MT81 (3)	MT81 (3)	MT42 (22)	MT114 (2)	MT81(4)	MT120(3)	MT29(38)
in each hospital	MT48 (29)	MT33 (3)	MT39 (2)	MT94(3)	MT79(3)			MT22 (2)		MT21 (2)
	MT52 (6)	MT6 (2)	MT72 (2)	MT47(3)	MT43(3)			MT45 (2)		
	MT53 (6)	MT12 (2)	MT78 (2)					MT75 (2)		
	MT49 (3)	MT18 (2)	MT104 (2)							
	MT96 (2)									
	MT110 (2)									

*MT, microsatellite typing.*

In hospital H01, isolates from 23 patients in surgical departments, three patients in ICU, and three patients in medicine departments exhibited the endemic genotype MT48 (total *n* = 29). The MT95 genotype was evident in isolates from 43 patients – 33 patients in surgical departments, seven patients in ICU, and three patients in medicine departments, whilst there were six isolates assigned to MT52 (four patients in surgical department and two patients in ICU) and six isolates assigned to MT53 (five patients in surgical departments and one patient in ICU). In this hospital, hence the endemic genotypes were found in different wards, though mainly in surgical wards. The MT48 first appeared in August 2015, persisted until December 2016, but appeared only one once more in June 2017. MT95, first emerged in December 2015, peaked in November 2016 (*n* = 5), and persisted until May 2017. These findings suggested the presence of potential outbreak in surgical wards, as most of the patients were infected within a short time span.

For 24 patients who had isolates cultured from blood and vascular catheter tips, the genotypes of each pair were identical in all 24 cases. Genotype data, however, from catheter isolates were not included in the phylogenetic analysis to avoid bias. The distribution of these 24 patients and the genotype data are as follows: Hospital H01 (MT48, *n* = 2; MT53, *n* = 1; MT95, *n* = 8), H02 (MT33, *n* = 1), H05 (MT43, *n* = 1; MT73, *n* = 1; MT81, *n* = 1), H06 (MT42, *n* = 3), and H10 (MT29, *n* = 4; MT68, *n* = 1; MT111, *n* = 1).

**FIGURE 2:**
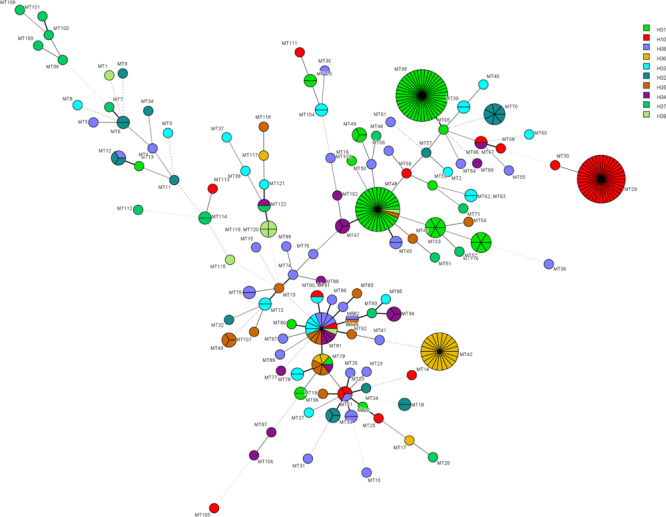
Minimum spanning tree showing the relationship of 122 MT types among 319 isolates distributed in ten hospitals. Each circle represents a unique genotype. The size of the circle corresponds to the number of isolates of the specific genotype. Different colors represent different hospitals. Fluconazole resistant isolates were distributed in genotypes MT15 (H08, *n* = 1), MT47 (H04, *n* = 3), MT48 (H09, *n* = 1), MT50 (H08, *n* = 1), MT70 (H02, *n* = 7), MT75 (H08, *n* = 2), and MT114 (H07, *n* = 1). 5-flucytosine NWT strains were distributed in genotypes MT45 (H08, *n* = 2), MT48 (H01, *n* = 29), MT52 (H01, *n* = 1), MT88 (H04, *n* = 1), MT96 (H01, *n* = 2), MT97 (H04, *n* = 1), MT106 (H04, *n* = 1).

The relationship between genotypes was shown in the minimum spanning tree in Figure 2. Figure 2 shows the genetic relationship according to different hospitals where most genotypes in each hospital are sporadically distributed. The endemic genotypes in hospital H01 were genetically closely related. The isolates from most cluster cases shared the same susceptibility pattern, except for endemic genotype MT52, one isolate from H01 showed NWT MICs to 5-flucytosine, while the remaining five isolates had WT MICs. In hospital H01, there were 29 isolates belonging to genotype MT 48, all which exhibited high MIC (>64 μg/mL) to 5-flucytosine. Of a total of 16 fluconazole-resistant isolates, seven were from hospital H02 (all genotype MT70), and three were from hospital H04 hospital (genotype MT47).

## Discussion

*Candida parapsilosis* has been reported to be a significant clinical pathogen, being able to form biofilms on central venous catheters and other medically implanted devices, and which can persist in the hospital environment. This last characteristic gives propensity for this fungus to cause nosocomial case clusters via carriage on hands of staff (Trofa et al., 2008; van Asbeck et al., 2009; Toth et al., 2019). In the present study, we used microsatellite genotyping method to study the molecular epidemiology of *C. parapsilosis*, cultured from patients with invasive fungal infections, as part of the national surveillance program CHIF-NET 16-17. We chose to use microsatellite typing as this method has emerged as a powerful tool to study the genetic relationship between *C. parapsilosis* isolates in the outbreak setting (Vaz et al., 2011; Diab-Elschahawi et al., 2012; Pulcrano et al., 2012; Reiss et al., 2012; Romeo et al., 2013; Delfino et al., 2014; Sabino et al., 2015; Wang et al., 2016; Badali et al., 2017; Magobo et al., 2017; Desnos-Ollivier et al., 2018).

Data for 319 patients were analyzed; of these 230 patients suffered from candidemia. The patients were mainly adults from surgical departments and ICUs, except for the patients in hospital H06 hospital (mainly NICU). By microsatellite typing, we uncovered 32 cluster cases in 10 hospitals during a period of 2 years. The timeline of cluster cases indicates that certain endemic genotypes have persisted in the hospital setting, and presumably were difficult to eradicate, consistent with previous reports (Escribano et al., 2013, 2018). Wang et al. (2016) previously reported details of a *C. parapsilosis* outbreak in China, where 97 isolates comprising only two clones were mainly obtained from the ICU and surgical wards. Other *C. parapsilosis* outbreaks in ICUs have been reported from countries such as Brazil and Turkey (Dizbay et al., 2008; Pinhati et al., 2016). Notably, in the present study, strains of the MT42 genotype were responsible for a 22-case cluster in the NICU of hospital H06 (Figure 1); previously, case clusters in NICUs have been uncommonly reported in China. *C. parapsilosis* has been recognized as a significant pathogen in NICUs worldwide (Toth et al., 2019). A meta-analysis of neonatal candidiasis by Pammi et al. (2013) showed that *C. parapsilosis* comprised 33.5% of all neonatal *Candida* infections, and was associated with 10% mortality. Not unexpectedly, in the 24 patients from whom *C. parapsilosis* isolates were cultured simultaneously from blood and catheter tips, the isolates all exhibited the same genotype, consistent with the widely accepted notion that vascular catheters often represent the source of candidemia and the meticulous catheter care is important for source control (Escribano et al., 2018).

Notably, there were fluconazole resistant isolates responsible for the case clusters in the present study, particularly in two hospitals (three isolates from patients in hospital H04 and seven from patients in H02 hospital). Others have reported clonal emergence or outbreaks of fluconazole-resistant isolates of *C. parapsilosis* (Pinhati et al., 2016; Thomaz et al., 2018; Singh et al., 2019). Hence such clusters should signal an alert to clinicians and microbiologists alike. Raghuram et al. have reported that fluconazole-resistant strains of *C. parapsilosis* may be more pathogenic than fluconazole-susceptible strain in liver transplant patients (Raghuram et al., 2012). In another report, a patient infected with azole-resistant *C. parapsilosis* died despite appropriate antifungal treatment (Zhang et al., 2015). Overall, however, only 5% of *C. parapsilosis* isolates were resistant to fluconazole in the present study compared with 3.9% in the SENTRY program (Pfaller et al., 2019). Despite being overall uncommon, resistance of *C. parapsilosis* to fluconazole has been increasing reported, and the data from SENTRY Program suggest that fluconazole-resistant *C. parapsilosis* could emerge in the presence of drug pressure during fluconazole treatment and prophylaxis, with subsequent transmission between patients in the hospital environment (Pfaller et al., 2019).

Of equal concern, in hospital H01, there were clonal clusters of a 5-flucytosine NWT *C. parapsilosis* strain (MT48, *n* = 29), indicating an unrecognized *C. parapsilosis* cluster case due to a flucytosine NWT isolate, another rare event in China. 5-flucytosien NWT strains have been reported for *Candida tropicalis* in France (Desnos-Ollivier et al., 2008). Xiao et al. (2015) reported in CHIF-NET 2010-12 that only 0.8% *C. parapsilosis* species complex were NWT to 5-flucytosine, and all isolates were inhibited at drug concentrations of ≤1 mg/L. In contrast, in the present study 11.6% isolates were NWT to 5-flucytosine, and were mainly distributed in hospital H01 with the MIC NWT isolates ≥64 mg/L. 5-flucytosine is typically used in combination with other antifungals. The combination of 5-flucytosine and azoles or amphotericin B remains primary treatment for *Candida* meningitis or endocarditis (Hoeprich et al., 1974; Faix, 1984). The emergence of 5-flucytosine NWT isolates in the outbreak setting poses the question of whether resistance may be transmitted between patients like fluconazole-resistant isolates.

There were some limitations to this study. First, information of clinical characteristics, risk factors, and outcomes of patients with *C. parapsilosis* infections and those associated with the cluster cases were not available. Second, the study was performed retrospectively, and we could not collect the isolates from the hospital environment or health care workers’ hands, thus it was not possible to identify the mode of transmission. Third, there was no data about patients transferring between different wards within the hospitals, as well as the rotation of healthcare workers between different departments, and therefore this limited the explanation as to why there were certain endemic genotypes existing in different areas within any one hospital. Further work is needed to investigate the clinical and microbiological consequences of the current cluster cases, especially in hospitals H01, H06, and H10.

Nonetheless the present study was instructive in that it brought to light, several nosocomial cluster cases of *C. parapsilosis* infection including those involving azole-resistant strains. Lack of awareness of healthcare-associated infections and transmission, particularly those caused by fungi, is common in hospitals in China. Increasing candidemia morbidity and emergence of azole-resistant *Candida* species serve to emphasize the attention required for effective management of *Candida* infections including strategies for infection control.

## Data Availability Statement

All datasets generated for this study are included in the article/Supplementary Material.

## Ethics Statement

The program was approved by the Human Research Ethics Committee of Peking Union Medical College Hospital (S-263).

## Author Contributions

LZ, S-YY, MX, T-SS, and Y-CX conceived and designed the experiments. LZ, S-YY, Y-TN, WK, S-MD, M-LZ, and GZ performed the experiments. LZ, MX, XH, and HW performed the data analysis and wrote the manuscript. M-YL, SC, and FK revised the manuscript critically for important intellectual content. All authors participated in the critical review of this manuscript.

## Conflict of Interest

The authors declare that the research was conducted in the absence of any commercial or financial relationships that could be construed as a potential conflict of interest.
